# Comparison of Clinical & Laboratory Standards Institute standards in antimicrobial susceptibility among the carbapenemase producing *Enterobacteriaceae*

**DOI:** 10.4155/fsoa-2017-0095

**Published:** 2017-10-05

**Authors:** Fidelis Enyinnaya, Patricia Cruz, Mark P Buttner, Chad Cross, David R Woodard

**Affiliations:** 1 Department of Environmental & Occupational Health, School of Community Health Sciences, University of Nevada, Las Vegas, 4505 S. Maryland Parkway, Box 453064, Las Vegas, NV 89154–3064, USA; 2 Department of Surgical Critical Care, Hartford Hospital, 80 Seymour Street Hartford, CT 06102, USA; 3 Department of Radiation Oncology, School of Medicine, University of Nevada, Las Vegas, 4505 S. Maryland Parkway, Box 453070, Las Vegas, NV 89154–3070, USA

**Keywords:** antibiotics, antimicrobial stewardship, carbapenem, carbapenem resistance, Clinical and Laboratory Standards Institute, *Klebsiella pneumoniae*

## Abstract

**Aim:**

Carbapenems are antibiotics reserved for treatment of severe infections. Carbapenem antimicrobial susceptibility testing profiles were determined in a population of *Klebsiella pneumoniae*, and their resistance assessed based on previous and current Clinical and Laboratory Standards Institute criteria.

**Materials & methods:**

Isolates were examined using an automated antimicrobial susceptibility testing method, and real time polymerase chain reaction to detect the resistance (*blaKPC*) gene.

**Results:**

The prevalence of *blaKPC* gene was 45/54 (83.3%). Five isolates that were susceptible under the previous criteria changed to nonsusceptible with the current standards. The overall difference in susceptibility between the standards was 8%.

**Conclusion:**

This study shows that the current Clinical and Laboratory Standards Institute criteria may not offer additional benefits in the fight against carbapenem-resistant *Enterobacteriaceae*.

## Lay abstract

Carbapenem-resistant *Enterobacteriaceae* (CRE) are a family of bacteria that can result in deadly infections because they are resistant to most antibiotics. We obtained 54 *Klebsiella pneumonia* carbapenemase isolates, a type of CRE, and determined which antibiotics were effective against them. Results showed that five isolates that were considered susceptible to an antibiotic under the previous criteria were now considered nonsusceptible with the current standards. This study shows that the current criteria may not offer additional benefits in the fight against CRE infections. Studies such as this one and prudent use of antibiotics should limit the spread of antibiotic resistance.

Carbapenem-resistant *Enterobacteriaceae* (CRE), an emerging threat to public health, belong to a family of microorganisms that are difficult to treat because they are highly resistant to antibiotics. The *Enterobacteriaceae* include common species, such as *Klebsiella* spp. and *Escherichia coli*. These bacteria can cause serious hospital- and community-acquired infections, such as bloodstream infections, wound infections, urinary tract infections and pneumonia. Carbapenems are a group of antibiotics that are usually reserved to treat serious infections, particularly those caused by microorganisms that are highly resistant to antibiotics. CRE infections can no longer be treated with carbapenem antibiotics as they are resistant to these antibiotics. The most commonly used method for detection of CRE is the measurement of minimum inhibitory concentration (MIC). MICs are important in diagnostic laboratories to confirm resistance of microorganisms to an antimicrobial agent and also to monitor the activity of new antimicrobial agents. The MIC is a quantitative measurement of antibiotic activity, and it is defined as the minimum concentration of an antibiotic that can inhibit visible microbial growth under normal conditions [1,2]. By convention, carbapenem resistance is defined as an MIC result of Intermediate (I) or Resistant (R) to carbapenems on any antibiotic susceptibility test [3]. According to the USA FDA, an organism is classified as resistant to carbapenem if the pure culture shows a microbial breakpoint with an MIC ≥4 μg/ml during susceptibility testing [4]. Microbial breakpoint refers to the MIC at which an organism is described as susceptible or resistant to a given antibiotic. It is important that the MIC of antibiotics be lower than their breakpoint.

The Clinical and Laboratory Standards Institute (CLSI) publishes updated editions of its antimicrobial susceptibility testing (AST) standards. These updated criteria are intended to detect emerging bacterial resistance patterns. Periodic updates of these guidelines are essential as bacteria acquire resistance to antibiotics over time, and using the most current knowledge ensures that infections are treated consistently and fosters good antimicrobial stewardship.

Automated AST systems have become the most common method of conducting susceptibility testing; these instruments measure the MIC of antibiotics using the CLSI guidelines in place at the time of the approval of the procedure by the FDA. The FDA considers nonsusceptibility to antibiotics as MIC results that are intermediate or resistant according to fixed breakpoints; in addition, the agency is responsible for approving the use of CLSI criteria with automated AST systems. There is a time lag between the updates identified in the CLSI documents and the FDA approval for the particular testing instrument, and this lag results in the need for supplementary confirmation of resistance. The lower MICs for carbapenem antibiotics established in 2012 by the CLSI have not been endorsed by the FDA. These lower carbapenem MICs remain unchanged in the most current version of the CLSI guidelines [5]. There are different mechanisms of resistance to carbapenems; and a common mechanism is the production of *Klebsiella pneumoniae* carbapenemase (KPC). This enzyme breaks down carbapenems making them ineffective, and was first identified in the USA in 2001. In 2009, CLSI published a recommendation that carbapenem susceptible *Enterobacteriaceae* with susceptible, but elevated MIC, or with a reduced disk diffusion zone, be tested for the presence of the carbapenemase enzyme using the Modified Hodge Test (MHT) [6]. In 2010, the CLSI officially changed the carbapenem resistance criteria to ensure that KPC-producing organisms were not misclassified. This change eliminated the need for secondary testing using the MHT. Therefore, a lower MIC for antibiotic resistance was established for CREs; these criteria were again revised in 2012 [4]. Diagnostic kit manufacturers and clinical laboratories must have their kits approved by the FDA before they can be used with patient isolates, and these recent changes have led to confusion [6].

This study compared the MIC results in a population of *Klebsiella pneumoniae* obtained from a regional microbiology laboratory using the S19 (2009) and the S22 (2012) CLSI standards [6]. As indicated previously, the S22 carbapenem MICs remain unchanged in the most current version of the CLSI guidelines, S27 [5]. A significant problem in the laboratory detection of CRE is the fact that some bacterial isolates carry the *blaKPC* gene, while demonstrating susceptibility but an elevated MIC. This means that some isolates producing carbapenemase may test susceptible to carbapenems, and the CREs will not be correctly identified, thus posing an infection prevention and clinical management conundrum. The objectives of this study were to determine the presence of the *blaKPC* gene, the carbapenem AST profiles of clinical isolates and the resistance rates based on the previous and current CLSI criteria.

## Materials & methods

### Test organisms

Pure cultures of clinical isolates suspected of being CRE were obtained from a local core-microbiology laboratory. The core laboratory performs microbiology analysis for over eight general acute care hospitals, and a variety of long-term care facilities in our metropolitan area, which should ensure a heterogeneous selection of specimens. Analyses of these organisms were done in the Emerging Diseases Laboratory at the University of Nevada, Las Vegas, USA. Quality control *Enterobacteriaceae* strains were obtained from the American Type Culture Collection (ATCC, VA, USA). Two of these ATCC organisms are carbapenem resistant and possess the *blaKPC* gene, while four of these strains are susceptible to carbapenems and do not possess the *blaKPC* gene.

Bacterial DNA was extracted using the MoBio PowerSoil DNA extraction kit (MoBio, CA, USA) according to the manufacturer's instructions with one exception; 200 μl of sample (instead of 0.25 g) was used for extraction. The DNA extract was eluted in 100 μl of the C_6_ buffer solution provided, and stored at -70°C until ready for use.

#### Template DNA concentration

After DNA extraction, the amount of DNA in each sample extract was measured with a Spectronic TM Genesys 10 BIO UV-Visible spectrophotometer (Thermo Fisher Scientific, MA, USA) using the nanoCell accessory – 0.2 mm path length. Briefly, 1.0 μl of the sample extract was used, after zeroing the spectrophotometer with the C6 solution from the PowerSoil DNA extraction kit. The absorbance was set at 260/280 nm, with 320 nm reference wavelength and 2500 dilution factor. Samples were measured in duplicate and DNA concentrations were expressed in ng/μl. All samples with DNA concentration of 21 ng/μl and above were diluted using Tris-EDTA (TE) buffer (Teknova, CA, USA) prior to the polymerase chain reaction (PCR) assay. This enabled the DNA in the PCR to have a concentration between 10 and 100 ng (Applied Biosystems, Thermo Fisher Scientific, MA, USA).

### Real-time PCR

The 7900 HT Fast PCR System (Applied Biosystems) was used for detection and amplification of the *blaKPC* gene. Primers and a fluorescent probe specific for the *blaKPC* gene were identified from the literature [7,8]. The master mix was prepared using TaqMan 1X Universal Master Mix, sterile Nuclease Free Water, 0.3 μM of forward primer -5′-GAT ACC ACG TTC CGT CTG G-3′, 0.3 μM of reverse primer -5′-GCA GGT TCC GGT TTT GTC TC-3′ and 0.2 μM of probe -6-carboxyfluorescein-5′-AGC GGC AGC AGT TTG TTG ATT G-3′-6 carboxytetramethylrhodamine. Primers were obtained from Eurofins MWG Operon (AL, USA)USA) and the probe was obtained from Applied Biosystems.

All PCR assay reactions were performed in duplicate, using a total volume of 25 μl, containing 5 μl of template DNA (i.e., 10–100 ng) and 20 μl of the master mix solution. Positive and negative controls were included with each PCR assay. Following amplification, the results were analysed on the PCR computer using the SDS ver. 3.0 software. Once amplification was completed, the level of amplification was reported by the software as the mean Cycle Threshold (C_T_) value of replicate samples. C_T_ refers to the PCR cycle at which fluorescence (i.e., amplification product) is first detected. A sample was considered positive by real-time PCR and possessed the *blaKPC* gene, if the C_T_ crossed the threshold before the PCR cycle of 40. A C_T_ value of 40 or undetermined represents no target DNA present. Samples that tested undetermined for the *blaKPC* gene were re-analysed using an internal positive control to determine if there were inhibitors present in the reaction.

### Internal positive control

A commercially available TaqMan exogenous Internal Positive Control (Applied Biosystems) was used to detect PCR inhibition and to rule out false negatives. The *blaKPC* PCR assay was tested with the internal positive control; thus absence, or a decrease in amplification of the internal positive control DNA in each duplex PCR indicated the presence of PCR inhibitors.

### AST & microbial identification

AST was conducted on all isolates using Gram-negative AST and identification cards for the Vitek 2^®^ Compact system (bioMerieux, NC, USA) following the manufacturer's protocol. The results of the susceptibility profile were analysed on the Vitek 2^®^ system computer using software version 5.04 (bioMerieux) in accordance with the FDA (previous CLSI breakpoints) and the current CLSI carbapenem susceptibility breakpoints (Table 1). The Advanced Expert Analysis^®^ was applied to our analysis.

**Table 1.** **Performance standards for antimicrobial susceptibility testing.**

**Agent**	**Previous breakpoints M100-S19^†^ MIC (μg/ml)**			**Current breakpoints M100–27^‡^ MIC (μg/ml)**		

	***S***	***I***	***R***	***S***	***I***	***R***
Doripenem	–	–	–	≤1	2	≤4

Ertapenem	≤2	4	≥8	≤0.5	1	≥2

Imipenem	≤4	8	≥16	≤1	2	≥4

Meropenem	≤4	8	≥16	≤1	2	≥4

^†^2009 CLSI criteria [9].

^‡^2017 CLSI criteria [5].

I: Intermediate; MIC: Minimum inhibitory concentration; R: Resistant; S: Susceptible.

#### Statistical analysis

The carbapenem susceptibility of each isolate was determined using two different CLSI breakpoint standards (i.e., 2009 and 2017), and the proportion of susceptible isolates did not represent independent samples, thus traditional contingency table analyses were not appropriate. Therefore, data for the CRE isolates were subjected to a test of marginal homogeneity using counts of susceptibility (S) and resistance (R) as the outcome variable. That is, when comparing the two sets of standards, isolates could have one of four outcomes, namely: S→S, S→R, R→S, or R→R. Counts for these outcomes were analysed using the Stuart-Maxwell test [10,11] which is the generalization of the McNemar's test for a 2 × 2 table. Since some cells within the table had values of zero, it was important to transform the counts into usable data prior to analysis. This was accomplished by employing the ‘rule of three’ methodology proposed by Jovanovic and Levy [12], and Browne [13], which adds a very small fraction to each cell in the table; this makes each cell count usable without sacrificing the precision of the estimate. We employed the ‘irr’ package in R^®^ (v. 3.2.1) and report exact p-values for two sets of tests:  determination of statistical differences between S and R isolates, and determination of statistical differences between the carbapenem profiles amongst *blaKPC* gene-positive isolates when using the 2009 versus 2017 breakpoint standards. It should be noted that data for Doripenem were not analysed owing to the absence of an established cut-point for 2009.

## Results

Fifty-six isolates were received and subcultured on TSA, and incubated for 24–48 h. Of these, one did not grow after various attempts to culture it. Two additional isolates had the same identification number; therefore, one of these was not analysed. In total, 54 of 56 isolates received were subjected to analysis. These isolates were recovered from urine (catheter, and clean catch; n = 26), sputum (n = 15), bronchiole (n = 1), wound (n = 5), abscess (n = 1), blood (n = 2), abdominal fluid (n = 1) and unspecified location (n = 3). All isolates with mean C_T_ values <40 were regarded as positive, and considered to harbor the *blaKPC* gene. Known *blaKPC* gene negative ATCC strains tested PCR negative (undetermined) for the presence of the *blaKPC* gene (data not shown).

All bacterial isolates from the reference laboratory were verified using the Vitek 2^®^ Compact instrument. Microbial identification carried out on CRE isolates using the Vitek 2 ID card No. 21341 identified the following organisms with at least 94% confidence: *Klebsiella pneumonia* (n = 46), *Escherichia coli* (n = 2), *Enterobacter aerogenes* (n = 2), *Citrobacter freundii* (n = 2), *Acinetobacter baumannii* (n = 1) and *Proteus mirabilis* (n = 1).

Ten of the 54 isolates produced negative (undetermined) results and were regarded as negative for the *blaKPC* gene. Additional PCR analyses performed with the Internal Positive Control on these negative isolates showed inhibitors in several *blaKPC* gene negative isolates. Subsequently, serial dilution (1:10 and 1:100) of the inhibited samples resulted in a positive *blaKPC* gene by PCR. The prevalence rate of the *blaKPC* gene among the 54 suspected CRE isolates was 83.3% (45 of 54). Isolates identified as *Klebsiella pneumoniae* comprised the majority of our CRE isolates, and 97.8% (45/46) of these had the *blaKPC* gene.

Table 2 shows the differences in susceptibility of our organisms comparing the two different breakpoints. For example, transitioning to the current CLSI criteria for Ertapenem would result in a change in resistance rate from 88.7 to 90.6%. Transitioning to the current criteria would result in a change in resistance rate from 85.2 to 90.7% when testing against Imipenem. For Meropenem, a change in resistance rates from 87.0 to 90.7% was observed. However, statistical differences were not demonstrated for Ertapenem (χ^2^ = 0.90; p = 0.342), Imipenem (χ^2^ = 2.90; p = 0.089), or Meropenem (χ^2^ = 1.90; p = 0.168).

**Table 2.** **Individual carbapenem susceptibility among all Carbapenem-resistant *Enterobacteriaceae* isolates analyzed.**

**Individual carbapenems**	**Previous breakpoints M100-S19^†^**				**Current breakpoints M100–27^‡^**			

	**Susceptible**		**Nonsusceptible**		**Susceptible**		**Nonsusceptible**	

	***n***	***%***	***n***	***%***	***n***	***%***	***n***	***%***
Ertapenem^§^	6	11.3	47	88.7	5	9.4	48	90.6

Imipenem	8	14.8	46	85.2	5	9.3	49	90.7

Doripenem^¶^	nd	nd	nd	nd	5	9.6	47	90.4

Meropenem	7	13.0	47	87.0	5	9.3	49	90.7

^†^2009 CLSI criteria [9].

^‡^2017 CLSI criteria [5].

^§^Ertapenem use was not indicated for one isolate.

^¶^Doripenem use was not indicated for two isolates.

CRE: Carbapenem resistant *Enterobacteriaceae*; nd: Doripenem interpretation was not defined in the previous breakpoints.

Five isolates changed from susceptible to nonsusceptible for at least one carbapenem when using the current criteria (Table 3). Four of these were *blaKPC* gene negative (Table 4). Carbapenemase (metallo- or KPC) were implicated as the mechanism of resistance in four isolates. Other resistance mechanisms implicated included Extended Spectrum Beta- lactamase, Amp, and impermeability. Specifically these isolates included three non-*Klebsiella* species and two *Klebsiella pneumoniae*. Statistical differences in susceptibility profiles amongst isolates with the *blaKPC* gene were not demonstrated for Ertapenem (χ^2^ = 0.64; p = 0.423), Imipenem (χ^2^ = 1.59; p = 0.207) or Meropenem (χ^2^ = 1.85; p = 0.174).

**Table 3.** **Individual carbapenem susceptibility among *blaKPC* gene negative isolates.**

**Individual carbapenems**	**Previous breakpoints M100-S19^†^**		**Current breakpoints M100–27^‡^**	

	**Susceptible**	**Nonsusceptible**	**Susceptible**	**Nonsusceptible**
Ertapenem	6	2	5	3

Imipenem	7	2	5	4

Doripenem	ND	ND	4	3

Meropenem	7	2	5	4

^†^2009 CLSI criteria [9].

^‡^2017 CLSI criteria [5].

ND: Doripenem interpretation was not defined in the previous breakpoint.

**Table 4.** **Susceptibility changes (susceptible to nonsusceptible) between the previous Clinical and Laboratory Standards Institute criteria and the current criteria among *blaKPC* genes (negative and positive).**

**Individual carbapenems^†^**	***blaKPC* gene negative**	***blaKPC* gene positive**	**Total number of changes**
Ertapenem	1	0	1

Imipenem	2	1	3

Meropenem	2	0	2

^†^Doripenem susceptibilities were not compared.

When testing our organisms using the AST Cards (GN69 and XN06, bioMerieux) (Table 5), there was a demonstrable, but statistically insignificant, change in the susceptibility observed when the organisms possessed a carbapenemase (metallo- or KPC; 83.3–90.7%; p = 0.150).

**Table 5.** **Carbapenem resistance phenotypes implicated in all isolates (n = 54).**

**Carbapenem resistance phenotype^†^**	**GN69 AST card (n; percentage)**	**XN06 AST card (n; percentage)**
Extended spectrum beta-lactamase	47 (87.0%)	47 (87.0%)

Impermeability (carbapenems and cephamycins)	46 (85.2%)	48 (88.8%)

Carbapenemase (metallo- or KPC)	45 (83.3%)	49 (90.7%)

Penicillinase (acquired or wild-type)	2 (3.7%)	1 (1.9%)

High level AmpC (HL-CASE)	2 (3.7%)	1 (1.9%)

Inhibitor-resistant PASE (IRT or OXA)	0 (0%)	1(1.9%)

^†^Most isolates exhibited more than one phenotype

AST: antimicrobial susceptibility testing; HL-CASE: High-level AmpC B-lactamase; IRT: Inhibitor-resistant TEM B-lactamases; KPC: *Klebsiella pneumoniae* carbapenemase; OXA: Oxacillinase group of B-lactamases.

## Discussion

The CLSI regularly, and most recently in 2017, publishes updated editions of its AST standards. The updated criteria are intended to respond to the emerging bacterial resistance patterns reported by clinical microbiology laboratories. One area of concern is the relative definitions of susceptible versus nonsusceptible between the FDA and the CLSI. In 2012, CLSI lowered its threshold for nonsusceptible CRE resistance; however, the FDA did not endorse this change, and thus there was discordance in the reporting of the susceptibility of some bacteria to the carbapenem antibiotics. The lower threshold established in 2012 for nonsusceptible CRE resistance remains unchanged in the most current version of the CLSI guidelines [5].

In this study, the prevalence of the *blaKPC* gene was 83.3% amongst suspected CRE isolates from different healthcare centers. Isolates identified as *Klebsiella pneumoniae* comprised the majority of our CRE isolates, and 97.8% of these had the *blaKPC* gene. These findings are similar to those of other studies in the USA reporting that the *blaKPC* gene is the most commonly implicated gene in carbapenem resistance amongst the *Enterobacteriaceae* [9].

The CLSI and the Centers for Disease Control and Prevention recommended that CRE isolates with antibiotic resistant profiles or elevated, but susceptible profiles, be confirmed with the Modified Hodge Test [14]. This recommendation has since been revised. Our results imply that the current CLSI criteria may not offer additional benefits in the management of CRE infections. These results are similar to others reported in the literature that showed either no change between the two breakpoints or unnecessary increase in the estimation of carbapenem resistance [15].

Because the quantity of antibiotics used on patients and the development of resistance are directly proportional, increased reports of *Enterobacteriaceae* resistant to carbapenems and reduced MIC breakpoints will increase the number of *Enterobacteriaceae* demonstrated to be resistant to at least one agent in any antimicrobial category. Therefore, clinicians potentially could prescribe increased doses of carbapenems or other antimicrobial classes, which may lead to the development of more resistance [16]. This may be particularly important in the nonhospital (e.g., nursing home) setting.

We also identified a potential for under-reporting of resistance when using the AST cards on the Vitek when the organisms possess a carbapenemase. In our small sample, the difference approached 8%. Given that this particular class of organism has the potential to occur in clones and that this organism may also be found in closed populations (such as long-term care facilities, and intensive care units), it has the potential for grave consequences in the arena of infection prevention and clinical management of infected patients [17].

The number of isolates included in this study was limited; however, our pilot study did provide data that can be utilized in further studies. Molecular detection of the *blaKPC* gene was only positive in isolates that express the *blaKPC* gene and may have underestimated the presence of other resistance mechanisms implicated in CRE infections, such as the MBL, the OXA, and the AmpC enzymes in *blaKPC* gene negative isolates. A limitation of this research was the exact mechanism for resistance in the isolates was not determined. However, this was outside the scope of the study. Because *Klebsiella pneumoniae* carbapenemase is the most commonly implicated carbapenemase in the USA, in a resource limited setting, targeting the *blaKPC* gene will be a more efficient way to detect and confirm carbapenem resistance. We did not perform the MHT as confirmation for carbapenemase activity in our CRE isolates; however, we were able to use a PCR-based assay as an alternative to verify the presence of the *blaKPC* gene.

The use of molecular testing did provide useful information regarding the prevalence of the *blaKPC* gene, and this technology appears to provide a more precise and timely method for determination of resistance in the *Enterobacteriaceae*. The PCR methodology also provides an advantage to the clinician in terms of time required to produce a true picture of the susceptibility pattern of an organism. This is consistent with the findings of Endimiani *et al*. [18]. Accurate and timely identification and reporting of carbapenemases amongst this class of microorganisms are essential in the implementation of the CDC's CRE toolkit [19]. A research question that has yet to be fully addressed is the prevalence of KPC occurring in the long-term acute care hospital and similar institutions when compared with the general acute care hospital and the epidemiological pattern of resistance across Nevada as well as nationally.

## Conclusion

The results from this study demonstrated the prevalence of the *blaKPC* gene and determined antimicrobial susceptibility profiles among CRE isolates in a discrete population. Five isolates that were susceptible under the previous criteria changed to nonsusceptible with the current standards. The overall difference in susceptibility between the standards was 8%.

Our results imply that the current CLSI criteria may not offer additional benefits in the fight against CRE. The mis-characterization of antimicrobial susceptibility has significant consequences in the management of infections caused by these pathogens; this could cause under reporting of susceptibility and potential treatment failures or delay in implementation of effective therapy.

## Future perspective

To ensure proper detection of emerging resistance, species-related zones of inhibition or MIC breakpoints should be published as a means to targeted control of CRE. Future studies should include a real-time PCR assay that is capable of detecting multiple resistance genes to provide rapid and accurate detection of carbapenem resistance. Timely intervention, such as good infection control practices, rapid detection and prudent use of antibiotics will ensure that the spread of carbapenem resistance among organisms is kept under control.

**Summary points**

- The prevalence of the *blaKPC* gene was 83.3% (45/54) among suspected carbapenem-resistant *Enterobacteriaceae* (CRE) isolates from a local core microbiology laboratory.
- Isolates identified as *Klebsiella pneumoniae* comprised the majority of our CRE isolates, and 97.8% (45/46) of these had the *blaKPC* gene.
- Polymerase chain reaction (PCR) inhibitors were observed in pure cultures of our isolates, and resulted in false-negative results. Serial dilution (1:10 and 1:100) of the inhibited samples resulted in a positive *blaKPC* gene by PCR.
- Molecular detection of the *blaKPC* gene was only positive in isolates that possess the *KPC* gene and thus may have underestimated the presence of other resistance mechanisms implicated in CRE infections, such as the MBL, the OXA, and the AmpC enzymes in *blaKPC* gene negative isolates.
- We identified a potential for under-reporting of resistance when using the antimicrobial susceptibility testing cards on the Vitek when the organisms possess a carbapenemase.
- Our results imply that the current Clinical Laboratory Standards Institute criteria may not offer additional benefits in the fight against CRE infections.
- Increased reports of *Enterobacteriaceae* resistant to carbapenems and reduced minimum inhibitory concentration breakpoints will increase the number of *Enterobacteriaceae* determined to be resistant to at least one agent in any antimicrobial category. Therefore, clinicians potentially could prescribe increased doses of carbapenems or other antimicrobial classes, which may lead to the development of more resistance.
- Research questions that have yet to be fully addressed are the prevalence of KPC occurring in the long-term acute care hospital compared with the general acute care hospital, and the epidemiological pattern of resistance across Nevada as well as nationally.
